# Inhibition of SREBP-1 Activation by a Novel Small-Molecule Inhibitor Enhances the Sensitivity of Hepatocellular Carcinoma Tissue to Radiofrequency Ablation

**DOI:** 10.3389/fonc.2021.796152

**Published:** 2021-11-26

**Authors:** Xiao-zheng Zou, Jun-feng Hao, Xiu-hua Zhou

**Affiliations:** ^1^ Department of Critical Care Medicine, The Fourth Affiliated Hospital of China Medical University, Shenyang City, China; ^2^ Department of Nephrology, Affiliated Hospital of Guangdong Medical University/Institute of Nephrology and Zhanjiang Key Laboratory of Prevention and Management of Chronic Kidney Disease, Guangdong Medical University, Zhanjiang City, China

**Keywords:** hepatocellular carcinoma, sterol regulatory element binding protein-1, radiofrequency ablation, aerobic glycolysis, small-molecule inhibitor, RFA sensitization

## Abstract

Radiofrequency ablation (RFA) is an important strategy for treatment of advanced hepatocellular carcinoma (HCC). However, the prognostic indicators of RFA therapy are not known, and there are few strategies for RFA sensitization. The transcription factor sterol regulatory element binding protein 1 (SREBP)-1 regulates fatty-acid synthesis but also promotes the proliferation or metastasis of HCC cells. Here, the clinical importance of SREBP-1 and potential application of knockdown of SREBP-1 expression in RFA of advanced HCC was elucidated. In patients with advanced HCC receiving RFA, a high level of endogenous SREBP-1 expression correlated to poor survival. Inhibition of SREBP-1 activation using a novel small-molecule inhibitor, SI-1, not only inhibited the aerobic glycolysis of HCC cells, it also enhanced the antitumor effects of RFA on xenograft tumors. Overall, our results: (i) revealed the correlation between SREBP-1 and HCC severity; (ii) indicated that inhibition of SREBP-1 activation could be a promising approach for treatment of advanced HCC.

## Introduction

Radiofrequency ablation (RFA) is an important local therapeutic strategy for advanced hepatocellular carcinoma (HCC) (1–3). For patients with advanced HCC who are not suitable for resection, RFA can damage HCC tissue accurately and minimize damage to healthy liver tissue (4–6). RFA is considered to have several advantages, but its application can pose two major problems. First, incomplete RFA can change the characteristics of HCC tissue (e.g., induce epithelial–mesenchymal transition (EMT) of HCC tissue) and induce the recurrence and metastasis of HCC (7, 8). Second, the prognosis of HCC patients after RFA is not known (9–11). Hence, research on RFA against HCC is important.

A very high uptake of glucose mediates the aberrant metabolism of HCC cells (12, 13). This feature participates in regulation of the physical processes HCC cells, including proliferation, metastasis, EMT, or the resistance of HCC cells to antitumor therapies (14–16). Therefore, aberrant glucose metabolism in HCC cells could be a useful target to enhance the sensitivity of HCC to antitumor therapies.

Increasing evidence has revealed that lipid metabolism plays an important part in the high capability of HCC cells to uptake glucose (17–19). Almost 60% of the glucose taken up by HCC cells is used for the synthesis of fatty acids (17–19). Sterol regulatory element binding protein (SREBP)-1 is the most important transcription factor in lipid metabolism (20–22). In HCC cells, SREBP-1 mediates the transcription of genes related to the syntheses of fatty acids and triglycerides (17–19). Inhibition of activation of SREBP-1 *via* small-molecule inhibitors or small interfering (si)RNAs of SREBP-1 not only reduces the syntheses of fatty acids and triglycerides, it also inhibits glucose uptake (23, 24). In the present work, the results indicated that SREBP-1 is a promising target for RFA treatment. Inhibiting of SREBP-1 *via* its small molecular inhibitor SI-1 (SREBP-1 inhibitor) enhanced the sensitivity of HCC cells to RFA. This study not only expands our understanding of SREBP-1, but also provides new enlightenment for RFA treatment of HCC.

## Materials and Methods

### Clinical Samples and Cell Lines

Eighty-one patients with advanced HCC who underwent RFA were included in the present work. The protein samples extracted from these clinical specimens were provided by Professor Hui Xie (Beijing 302^nd^ Hospital, Beijing, China), as described previously (25). These tissue specimens have been prepared as samples for SDS-PAGE. The baseline information of patients were shown as **Supplemental Table 1**. The actual situation is: the sample used for western blot detection, the character is the sample extracted by SDS-PAGE loading buffer (Use PCR tubes for aliquoting), stored at -80 degrees; it is frozen and mailed by dry ice preservation and ultra-low temperature.

For cell lines, L-02 (hepatic non-tumor cell line) and HCC cells lines were gifts from Prof. and Dr. Fan Yin in the Department of Oncology, The Second Medical Center & National Clinical Research Center of Geriatric Disease, Chinese PLA General Hospital, Beijing, China and employed as described in the previous publication (26). They were cultured in Dulbecco’s modified Eagle’s medium containing 10% fetal bovine serum at 37°C in an atmosphere of 5% CO_2_.

In the presence work, all studies do not involve clinical trials and do not directly use patient-sourced materials. Possible human related may include some biochemical reagents and cell lines, which have been approved by the Fourth Affiliated Hospital of China Medical University.

### Western Blotting and Survival Analyses

SREBP-1 expression in clinical specimens was measured by western blotting. The expression level of SREBP-1 was examined in the total protein samples extracted from the clinical specimens. The antibodies used against SREBP-1 and methods of western blotting were as described in our previous publication. SREBP-1 expression was measured by quantitative analysis of western blots *via* images J (27, 28). Patients were divided into a SREBP-1 high-level group or SREBP-1 low-level group according to the median value of SREBP-1. Various parameters associated with SREBP-1 expression were assessed: time to disease progression (TTP) post-RFA; overall survival (OS); clinical efficacy response (CER)/overall response rate [i.e., complete response (CR) + partial response (PR)]; disease-control rate (DCR) [i.e., CR + PR + stable disease (SD)] (29, 30).

### Small-Molecule Inhibitor of SREBP-1

A small-molecule inhibitor of SREBP-1, 1-(4-bromophenyl)-3-(pyridin-3-yl)urea, was chemically synthesized (^1^H NMR (400 MHz, DMSO-*d*_6_) δ(ppm): 8.90 (d, *J* = 29.0 Hz, 2H), 8.58 (d, *J* = 2.6 Hz, 1H), 8.17 (dd, *J* = 4.7, 1.8 Hz, 1H), 7.98–7.87 (m, 1H), 7.43 (d, *J* = 2.1 Hz, 4H), 7.29 [(dd, *J* = 8.2, 4.7 Hz, 1H), MS *m/z* (M + H)^+^: 292.41] was also gifts from Prof. and Dr. Fan Yin in the Department of Oncology, The Second Medical Center & National Clinical Research Center of Geriatric Disease, Chinese PLA General Hospital, Beijing, China. The SREBP-1 inhibitors fatostatin (catalog number: S9785) and betulin (S4754) were purchased from Selleck Chemicals (Houston, TX, USA). The powder of SI-1, fatostatin, or betulin was prepared as described in the previous publications (31–34). HCC cells were cultured and treated with the indicated concentrations of agents, and harvested for real-time reverse transcription-quantitative polymerase chain reaction (RT-qPCR), 3-(4,5-Dimethylthiazol-2-yl)-2,5-diphenyltetrazolium bromide (MTT) assay, or biochemical analyses.

### Subcutaneous Tumor Model and RFA

For the animal experiments (the usage of nude mice) were reviewed and approved by the Institutional Animal Care and Use Committee (IACUC) of China Medical University. MHCC97-H cells were cultured and injected into nude mice (4–6 weeks; Si-Bei-Fu Corporation, Beijing, China) to form subcutaneous tumors (35, 36). The volume of each subcutaneous tumor was measured as width × width × length/2. When the volume of the subcutaneous tumor formed by MHCC97-H cells reached 500 mm^3^, nude mice underwent treatment (SI-1, RFA, or RFA + SI-1) (37).

For the RFA group, the subcutaneous tumor underwent RFA using a thyroid-ablation needle (UniBlate 700-103587 17G; RITA, Crystal Lake, IL, USA) at the indicated temperature (50°C, 55°C, 60°C, or 65°C) for 2 min. For the SI-1 group, nude mice received SI-1 (5, 2, 1, 0.5, or 0.2 mg/kg) *via* the oral route. For the RFA + SI-1 group, nude mice underwent RFA (50°C for 2 min) followed by administration of SI-1 (1 mg/kg bodyweight) (37). Tumor weights were measured using a precision balance.

### Real-Time RT-qPCR

MHCC97-H cells were cultured and treated with the indicated concentration (30, 10, 3, 1, 0.03, 0.01, or 0.003 μmol/L) of SI-1, botulin, or fatostatin. Tumor tissues were harvested for the subcutaneous tumor model (35, 36). The total RNA of cells or tumor tissues was extracted and reverse-transcribed into complimentary (c)DNA according to manufacturer (Thermo Fisher Scientific, Waltham, MA, USA) instructions and the methods described in our previous publications. cDNA samples also underwent real-time RT-qPCR according to a system from Thermo Fisher Scientific (38–40). The primers used in the qPCR were: (1) ACC, Forward Sequence, 5’-TTCACTCCACCT TGTCAGCGGA-3’; Reverse Sequence 5’-GTCAGAGAAGCAGCCCATCACT-3’; (2) ACLY, Forward Sequence, 5’-GCTCTGCCTA TGACAGCACCAT-3’; Reverse Sequence, 5’-GTCCGATGATGGTCACTCCCTT-3’; (3) FASN, Forward Sequence, 5-TTCTACGGCTCCACGCTCTTCC-3’; Reverse Sequence, 5’-GAAGAGTCTTC GTCAGCCAGGA-3’; (4) ACS, Forward Sequence, 5’-ATCAGGCTGCTCATGGATGACC-3’; Reverse Sequence, 5’-AGTCCAAGAGCCATC GCTTCAG-3’; (5) GLUT1, Forward Sequence, 5’-TTGCAGGCTTCTCCAACTGGAC-3’; Reverse Sequence, 5’-CAGAACCAGGAGCAC AGTGAAG-3’; (6) LDHA, Forward Sequence, 5’-GGATCTCCAACATGGCAGCCTT-3’; Reverse Sequence, 5’-AGACGGCTTTCTCCCT CTTGCT-3’; (7) HIF1α, Forward Sequence, 5’-TATGAGCCAGAAGAACTTTTAGGC-3’; Reverse Sequence, 5’-CACCTCTTTTGGCAA GCATCCTG-3’; (8) EPAS-1, Forward Sequence, 5’-CTGTGTCTGAGAAGAGTAACTTCC-3’; Reverse Sequence, 5’-TTGCCATAGGCTG AGGACTCCT-3’; (9) N-cadherin, Forward Sequence, 5’-CCTCCAGAGTTTACTGC CATGAC-3’; Reverse Sequence, 5’-GTAGGATCTCCGCC ACTGATTC-3’; (10) Vimentin, Forward Sequence, 5’-AGGCAAAGCAGGAGTCCACTGA-3’; Reverse Sequence, 5’-ATCTGGCGTTCCAGGGACTCAT-3’.

### Biochemical Analyses

Assays for measurement of glycolytic activity were carried out in HCC cells and tumor tissues (26, 41–43). We used kits for glucose uptake (colorimetric; ab136955; Abcam, Cambridge, UK), lactate (Lactate-Glo™; Promega, Fitchburg, WI, USA), adenosine triphosphate (colorimetric/fluorometric; ab83355; Abcam), and lactate dehydrogenase (LDH; MAK066; Sigma–Aldrich, Saint Louis, MO, USA). The results are shown as heatmaps following the methods described by Zhou et al., 2020 (44).

### Statistical Analyses

SPSS 9.0 (IBM, Armonk, NY, USA) was used for statistical analyses. Origin 6.0 (OriginLab, Northampton, MA, USA) was employed to calculate the half-maximal inhibitory concentration (IC_50_) of agents (45, 46). The Student’s *t*-test (single-tail) was used to compare two categorical variables. P < 0.05 was considered significant.

## Results

### A High Level of Endogenous SREBP-1 Is Associated With a Poor Outcome After RFA

**Figure 1:**
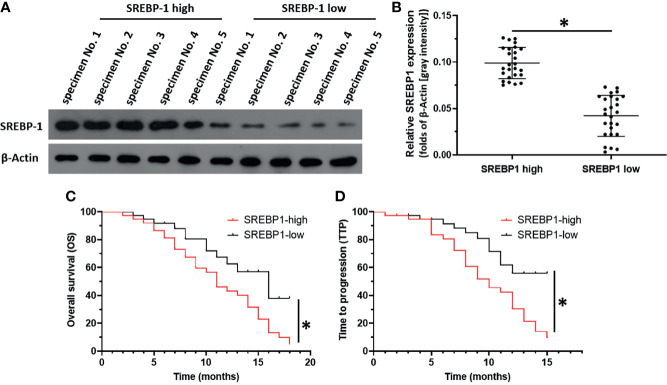
The correlation between SREBP-1’s expression with the prognosis of HCC patients post RFA. HCC tumor tissue obtained by coaxial puncture, using WB to detect the expression level of SREBP-1 in the tissue. **(A)** Representative images of WB results of 5 patients in the high expression group and 5 patients in the low expression group. **(B)** The results of WB were quantitatively analyzed by Image J software, and the relative expression level was calculated with the result of one of the specimens as unit 1. Divide patients into two groups based on the median relative expression level (SREBP-1 high group; SREBP-1 low group). **(C, D)** For SREBP-1 high group and SREBP-1 low group patients, combined with clinical data for survival analysis to determine the patient’s OS and TTP. *P < 0.05.

SREBP-1 expression in clinical specimens was measured to reveal the roles of this transcription factor in HCC and the effect of SREBP-1 on RFA when treating HCC. The prognosis of patients with a low SREBP-1 level (SREBP-1 low group, n=37) who received RFA was much better compared with that of patients with a high SREBP-1 level (SREBP-1 high group, n=37) who received RFA: the post-RFA TTP or OS of patients with a low SREBP-1 was much longer compared with that of patients with a high level of SREBP-1 (**Figure 1** and **Table 1**) (P<0.05). Moreover, patients with a low SREBP-1 level also had better CER (CR+PT) and DCR (CR+PR+SD) compared with those of patients with a high SREBP-1 **(** **Table 1** **)** (P<0.05). Therefore, a high level of SREBP-1 was associated with a poor prognosis of patients with advanced HCC who received RFA.

**Table 1:** SREBP1 expression and clinical outcome of sorafenib treatment.

	SREBP1 protein expression		P values
	High (n = 37)	Low (n = 37)	
TTP	10.0	12.0	0.005
	7.2-12.7 (M)	10.9-13.6 (M)	
OS	11.0	16.0	0.024
	8.7-13.3 (M)	10.7-21.3 (M)	
Overall response rate (CR+PR)	4 (10.81%)	17 (45.94%)	0.015
Disease control rate (CR+PR+SD)	16 (43.24%)	28 (75.67%)	0.027

TTP, time to progress; OS, overall survival; PR, partial remission; CR, complete remission; SD, stable of disease; M, months.

### SI-1 Inhibited SREBP-1 Activation in MHCC97-H Cells

**Figure 2:**
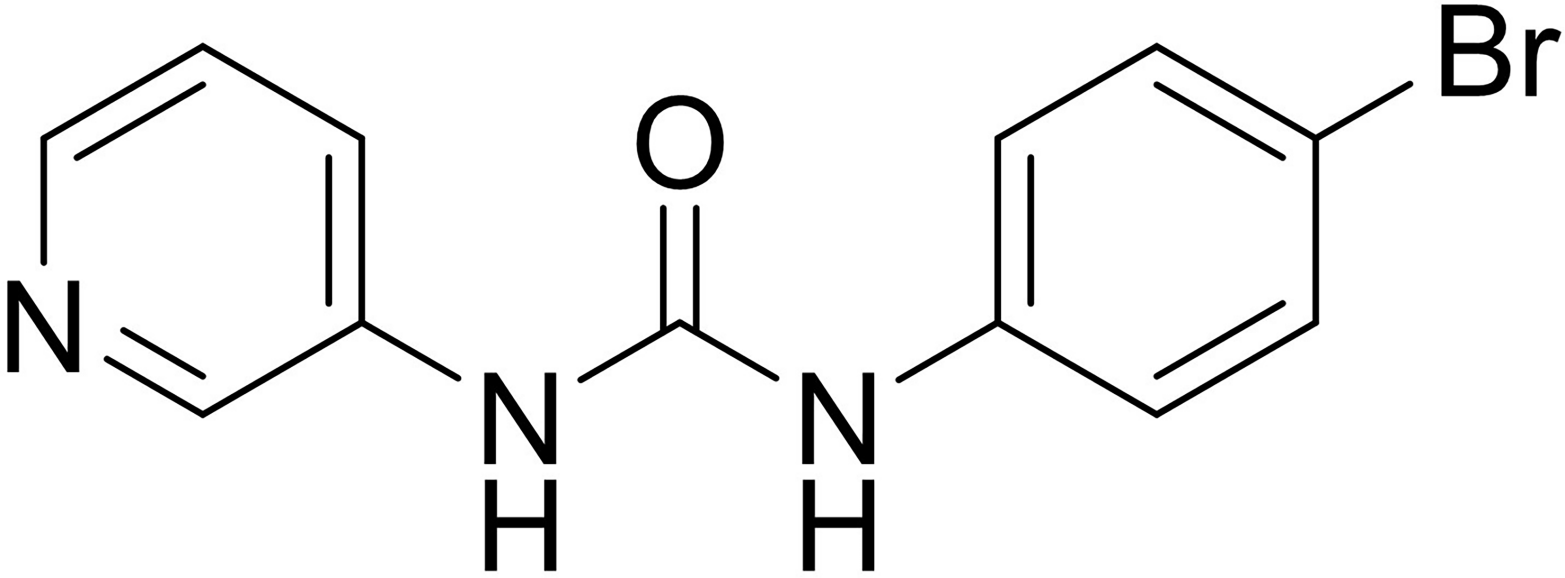
The structure of SI-1, a novel inhibitor of SREBP-1.

To explore the potential strategies targeting SREBP-1, a small-molecule inhibitor of SREBP-1, SI-1, was developed (**Figure 2**). In MHCC97-H cells (a typical HCC cell line with a high level of endogenous SREBP-1), SI-1 could inhibit SREBP-1 activation in a dose-dependent manner: SI-1 inhibited mRNA expression of the downstream genes of SREBP-1 (*ACC*, *ACLY*, *FASN* and *ACS*) (**Table 2**). Moreover, SI-1 inhibited Warburg effect-related features (glucose uptake, LDH activity, increased production of lactate and adenosine triphosphate), and expression of metabolism and hypoxic stress-related genes (*GLUT1*, *LDHA*) in the dose-dependent manner. EMT is an important regulator of the resistance of HCC cells to antitumor strategies. Hence, expression of the EMT-related indicators Twist, Snail, N-cadherin, and vimentin was examined. SI-1 inhibited EMT of MHCC97-H cells in a dose-dependent manner (**Table 2**). IC_50_ for SI-1 was much lower than that of the SREBP-1 inhibitors fatostatin or betulin (**Table 2**), which suggested that SI-1 was a more potent inhibitor of SREBP-1 activation than fatostatin or betulin.

**Table 2:** The activation of SI-1 compared with the Betulin or Fatostatin.

Factors	SI-1	Betulin	Fatostatin
	The *IC_50_* values (μmol/L)		
ACC	0.50 ± 0.01	1.48 ± 0.29	0.98 ± 0.15
ACLY	0.65 ± 0.08	1.27 ± 0.44	1.63 ± 0.53
FASN	0.34 ± 0.03	1.56 ± 0.46	1.03 ± 0.40
ACS	0.54 ± 0.13	1.08 ± 0.20	0.91 ± 0.15
GLUT1	0.72 ± 0.05	1.82 ± 0.24	1.53 ± 0.78
LDHA	0.61 ± 0.10	1.64 ± 0.62	1.15 ± 0.60
HIF-1α	0.88 ± 0.25	2.04 ± 0.65	1.17 ± 0.55
EPAS-1	0.78 ± 0.41	1.99 ± 0.11	1.33 ± 0.27
LDHA	0.79 ± 0.30	1.64 ± 0.33	1.20 ± 0.72
EPAS-1	0.86 ± 0.53	2.13 ± 0.25	1.59 ± 0.84
LDHA	0.26 ± 0.04	1.97 ± 0.78	1.35 ± 0.78
ATP	0.31 ± 0.02	1.82 ± 0.67	0.63 ± 0.11
Lactate	0.28 ± 0.08	1.71 ± 0.81	0.87 ± 0.05
Glucose uptake	0.47 ± 0.07	1.28 ± 0.37	0.45 ± 0.18
N-cadherin	0.79 ± 0.26	1.63 ± 0.25	0.91 ± 0.36
Vimentin	0.75 ± 0.32	2.06 ± 0.90	0.74 ± 0.09

### The Optimal Condition of SI-1 or RFA on Tumors Formed by MHCC97-H Cells

**Figure 3:**
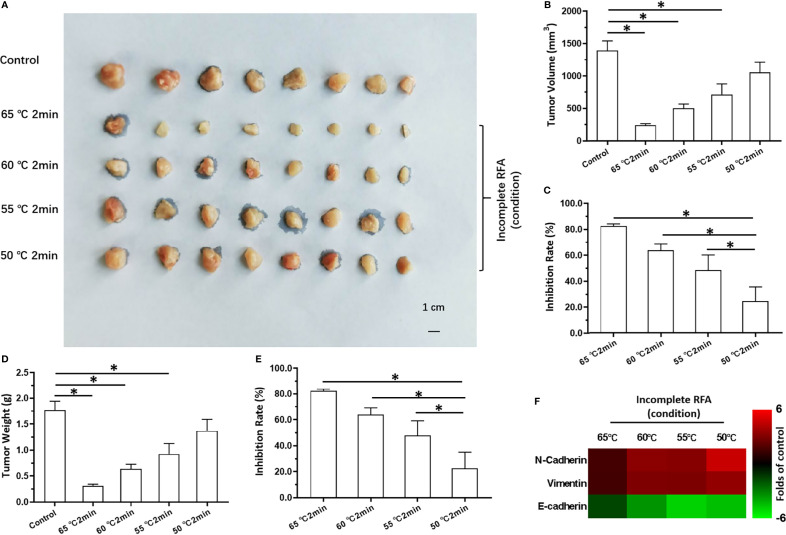
The *in vivo* antitumor activation of RFA on MHCC97-H cells. The MHCC97-H cells were cultured and injected into the subcutaneous position to form the subcutaneous tumor tissues. Then, the tumor tissues were performed by the RFA (65°C, 60°C, 55°C, 50°C) for 2min. The results were shown as images of tumors **(A)**, the tumor volumes **(B)**, the inhibitory rates according to the tumor volumes **(C)**, the tumor weights **(D)**, the inhibitory rates according to the tumor weights **(E)**, and the heat-map **(F)**. *P < 0.05.

The results stated above suggested SREBP-1 to be a promising target to enhance the effects of RFA against HCC. Therefore, to explore a therapeutic strategy combining SI-1 and RFA, the optimal condition of SI-1 or RFA was examined in subcutaneous tumors formed by MHCC97-H cells in nude mice. RFA carried out at 65°C for 2 min could lower the volume of HCC tissue significantly (**Figures 3A–E**). The RFA conditions of 60°C for 2 min or 55°C for 2 min also shrank HCC tissues (**Figures 3A–E**). RFA carried out at 50°C; for 2 min reduced HCC tissues only slightly but induced EMT of HCC cells in tumor tissues: increased expression of two mesenchymal markers (N-cadherin and vimentin) suppressed expression of an epithelial marker (E-cadherin) (**Figure 3F**). Therefore, RFA at 50°C for 2 min was chosen as the optimal condition of RFA for the next experiment.

**Figure 4:**
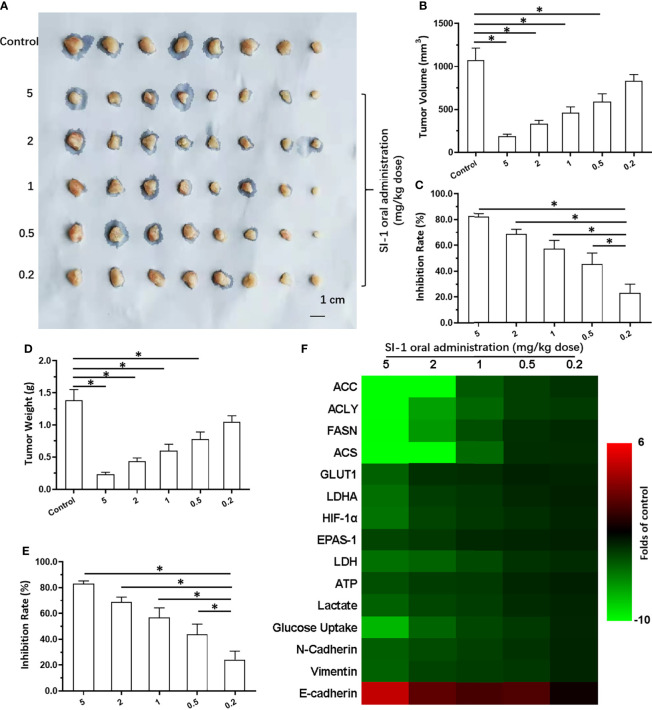
The *in vivo* antitumor activation of SI-1 on MHCC97-H cells. The MHCC97-H cells were cultured and injected into the subcutaneous position to form the subcutaneous tumor tissues. Then, the mice were received the SI-1 (5mg/kg, 2 mg/kg, 1 mg/kg, 0.5 mg/kg, 0.2 mg/kg) *via* oral administration. The results were shown as images of tumors **(A)**, the tumor volumes **(B)**, the inhibitory rates according to the tumor volumes **(C)**, the tumor weights **(D)**, the inhibitory rates according to the tumor weights **(E)**, and the heat-map **(F)**. *P < 0.05.

Oral administration of SI-1 inhibited the subcutaneous growth of MHCC97-H cells in a dose-dependent manner (**Figures 4A–E**). SI-1 (0.5–5 mg/kg) inhibited the subcutaneous growth of MHCC97-H cell (**Figures 4A–E**). SI-1 (0.2 mg/kg) could not exert antitumor activity but could significantly inhibit SREBP-1 activation (**Figure 4F**), the Warburg effect in HCC cells in tumor tissues (**Figure 4F**), or EMT (**Figure 4F**). Therefore, SI-1 (0.2mg/kg) was chosen for the next experiment.

### SI-1 Enhanced the Antitumor Effect of RFA Upon HCC

**Figure 5:**
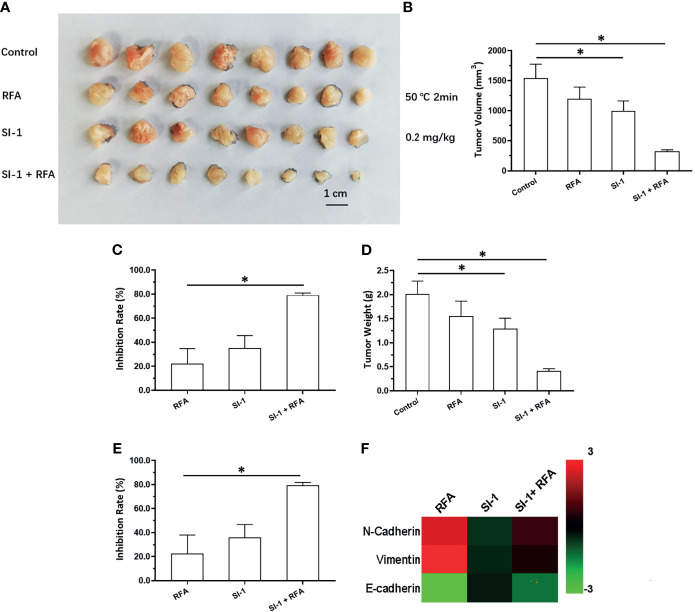
The *in vivo* antitumor activation of SI-1 with RFA on MHCC97-H cells. The MHCC97-H cells were cultured and injected into the subcutaneous position to form the subcutaneous tumor tissues. Then, the mice were received the RFA (50°C for 2min) or SI-1 (0.2 mg/kg) or RFA + SI-1. The results were shown as images of tumors **(A)**, the tumor volumes **(B)**, the inhibitory rates according to the tumor volumes **(C)**, the tumor weights **(D)**, the inhibitory rates according to the tumor weights **(E)**, and the heat-map **(F)**. *P < 0.05.

A combination of SI-1 (which represses SREBP-1 activation) and RFA on HCC was examined further. SI-1 (0.2 mg/kg) enhanced the antitumor effect of RFA (5°C for 2 min) (**Figure 5**). Use of SI-1 alone or RFA alone did not have a significant antitumor effect. The combination of SI-1 with RFA induced significant shrinkage of tumor volume (**Figures 5A–E**). SI-1 treatment also inhibited EMT of HCC cells in tumor tissues induced by RFA at 50°C for 2 min (**Figure 5F**). Therefore, SI-1 enhanced the antitumor effect of RFA upon HCC.

## Discussion

Human malignancies (especially HCC) are often characterized by anaerobic glycolysis/Warburg effect (47–49). These features aid energy generation for cellular proliferation and participate in alteration of the tumor microenvironment (49–51). Lipid metabolism is closely related to glucose metabolism (26). Hence, knockdown of SREBP-1 expression could inhibit glucose uptake or anaerobic glycolysis. Yin et al. showed that downregulation of SREBP-1 expression by betulin could enhance the sensitivity of HCC cells to molecular-targeted agents (26). Here, we revealed the novel roles of SREBP-1 in HCC regulation: SREBP-1 was related to the resistance of HCC to RFA, and knockdown of SREBP-1 expression was a promising approach to enhance the sensitivity of HCC cells to RFA. To inhibit SREBP-1 activation, a novel small-molecule inhibitor of SREBP-1, SI-1, was synthesized. We discovered that a high level of SREBP-1 in clinical specimens was correlated with a poor prognosis of HCC patients after RFA. SI-1 could inhibit SREBP-1 as well as the anaerobic glycolysis and EMT of HCC cells. Treatment with SI-1 enhanced the antitumor effect of RFA on HCC cells. Therefore, targeting SREBP-1 could be valuable for HCC treatment using RFA.

RFA is the most common therapeutic strategy for advanced-stage HCC (52–54). RFA is considered to damage tumor tissues/lesions and elicit little damage to normal liver tissues/adjacent liver tissue (52–54). Nevertheless, RFA has three main limitations. First, research has suggested that incomplete RFA may induce cellular stress and lead to pathologic changes (e.g., EMT) (55, 56). Second, the temperature used for RFA cannot be increased indefinitely otherwise liver injury and incomplete RFA will occur (55, 56). Third, incomplete RFA may also induce EMT of HCC cells in tissue to promote HCC recurrence (55, 56). We showed that SREBP-1 expression was closely related to the prognosis of HCC patients treated by RFA, and that use of small-molecule inhibitors of SREBP-1 could also inhibit metabolism-related EMT. It has been demonstrated that metabolic abnormalities (e.g., anaerobic glycolysis) are closely related to drug resistance (including resistance to molecular-targeted drugs) and stress/injury response (e.g., endoplasmic reticulum stress). Our study links RFA, lipid metabolism, and sugar metabolism in cancer cells. Incomplete RFA is an important factor in RFA research/treatment. We simulated incomplete RFA on nude mice. The RFA condition of 50°C for 2 min did not inhibit the subcutaneous growth of MHCC97-H cells in nude mice. The tumor volume shrank if RFA was supplemented with SI-1 treatment. Hence, knockdown of SREBP-1 expression may exert a sensitizing effect on RFA against HCC. Simultaneously, SI-1 may inhibit EMT in HCC cells induced by incomplete RFA (50°C for 2 min). Hence, SI-1 could be employed to avoid the problems caused by incomplete RFA and to achieve lower RFA intensity in combination therapy to achieve more robust anti-tumor activity.

**Figure 6:**
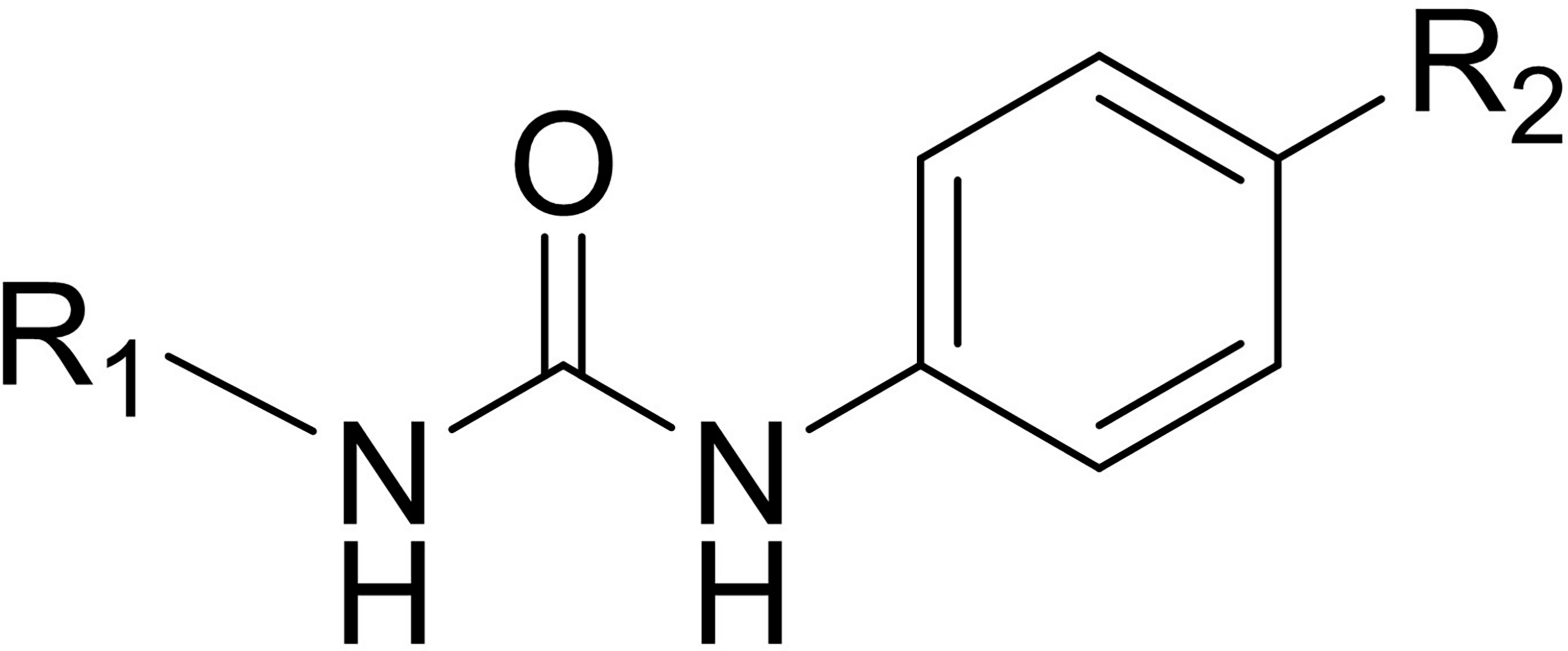
The core structure of SI-1. R1 and R2 refers to the position of the two substituents.

The structure of SI-1 that we synthesized was analyzed. **Figure 6** shows the chemical structure core of SI-1. In this structure, R1 can be C1–C6 alkyl, C3–C10 cycloalkyl, C1–C6 alkoxy, C1–C6 alkylthio, C3–C10 cycloalkoxy, or C1–C6 alkylene groups, alkenynyl heterocycle, heterocycloalkyl, substituted heterocycloalkyl, aromatic ring, aromatic heterocycle, or benzo aromatic heterocycle, wherein the C1–C6 alkyl, aromatic ring, aromatic heterocycle, benzene, and aromatic heterocyclic ring is unsubstituted or substituted by 1, 2, 3, 4 or 5 independently substituents selected from –F, –Cl, –Br, –I, nitro, hydroxyl, amino, cyano, C1–C6 alkylthio, C1–C6 alkyl, C1–C6 alkenyl, C1–C6 alkynyl, C1–C6 alkoxy, or aromatic groups. The chemical structure of SI-1 can be modified in future studies.

It has been confirmed that SREBP-1 is an important regulator of many liver diseases (57, 58). On the one hand, SREBP-1 plays an important role in metabolic diseases such as NAFLD (57, 59). On the other hand, SREBP-1 is also clearly regarded as a positive regulator of the occurrence and progression of HCC. In HCC cells, SREBP-1 can promote the proliferation, metastasis and invasion of HCC by promoting lipid metabolism and sugar metabolism (60–63). There are many reports on the molecular mechanism of SREBP-1 (19, 64–70). The activity of SREBP-1 is closely related to mTOR, c-MYC, AMPK and P38, and is also regulated by CAV1 (19, 64–70). These related studies have shown that SREBP-1 plays an important role in the occurrence and progression of HCC and is an ideal intervention target for HCC treatment.

Small molecule inhibitors are an ideal mode of action for specific targets (71–74). The existing SREBP-1 small molecule inhibitors are mainly Betulin, Pseudoprotodioscin and Fatostatin (75–77). In this study, a new SREBP-1 small molecule inhibitor SI-1 was prepared, and two existing inhibitors: Betulin and Fatostatin were used. The activity of SI-1 may be better than the two existing inhibitors. For these inhibitors, the main research report is to inhibit the activity of SREBP-1 as a tool in metabolism-related research. Betulin can exert anti-tumor activity in HCC (78–80). Pseudoprotodioscin and Fatostatin have been less studied in HCC, but it has also been clearly reported in other tumor types (81–84).

## Data Availability Statement

The original contributions presented in the study are included in the article/**Supplementary Material**. Further inquiries can be directed to the corresponding authors.

## Ethics Statement

The studies do not involve clinical trials, nor involve tissue specimens and other materials directly derived from patients. The usage of human related materials, including the protein samples for western blot or the cell lines were permitted by the Fourth Affiliated Hospital of China Medical University. The patients/participants provided their written informed consent to participate in this study. Written informed consent was obtained from the individual(s) for the publication of any potentially identifiable images or data included in this article.

## Author Contributions

X-hZ, X-zZ, and J-fH: concept, design, statistics, data collection, manuscript writing, final approval. X-hZ: design, statistics, data collection. X-zZ: concept, data collection. X-zZ and J-fH: statistics, manuscript writing. X-zZ: statistics, data collection. X-zZ and X-hZ: statistics, data collection. X-hZ: concept, design, statistics, data collection, manuscript writing, final approval. X-zZ and J-fH Finished the revised manuscript. All authors contributed to the article and approved the submitted version.

## Conflict of Interest

The authors declare that the research was conducted in the absence of any commercial or financial relationships that could be construed as a potential conflict of interest.

## Publisher’s Note

All claims expressed in this article are solely those of the authors and do not necessarily represent those of their affiliated organizations, or those of the publisher, the editors and the reviewers. Any product that may be evaluated in this article, or claim that may be made by its manufacturer, is not guaranteed or endorsed by the publisher.

## Supplementary Material

Supplementary Table 1 The baseline information of patients related to this work.
