# Supplementary material for: Inhibition of SREBP-1 Activation by a Novel Small-Molecule Inhibitor Enhances the Sensitivity of Hepatocellular Carcinoma Tissue to Radiofrequency Ablation
Source: Front Oncol. 2021 Nov 26;11:796152. doi: 10.3389/fonc.2021.796152 (PMC8660695; doi:10.3389/fonc.2021.796152)
Supplement: Supplementary Table 1 — The baseline information of patients related to this work. [file Table_1.doc]

Supplemental Table 1. The baseline information of patients related to this work

| **Characters** | **Num** (%) |
| --- | --- |
| Median age, years (range) | 49 (28-67) |
| Gender, male | 68 (83.95%) |
| HBV positive | 71 (87.65%) |
| HCV positive | 10 (12.35%) |
| AFP-Normal | 17 (20.99%) |
| AFP-Elevated | 64 (79.01%) |
| extrahepatic metastasis (%) | 32 (39.50%) |
| LN metastasis (%) | 39 (48.14%) |
| Portal vein invasion (%) | 43 (53.08%) |
| Chilg-Pugh A | 69 (85.18%) |
| Chilg-Pugh B | 12 (14.82%) |
| Median size of lsions, cm (range) | 1.4 (1-3.0) |
| Differentiation-Well | 10 (12.34%) |
| Differentiation-Moderate | 44 (54.32%) |
| Differentiation-Poor | 27 (33.33%) |
